# The Relationship Among Gastrointestinal Symptoms, Problem Behaviors, and Internalizing Symptoms in Children and Adolescents With Autism Spectrum Disorder

**DOI:** 10.3389/fpsyt.2019.00194

**Published:** 2019-04-09

**Authors:** Bradley J. Ferguson, Kristen Dovgan, Nicole Takahashi, David Q. Beversdorf

**Affiliations:** ^1^Department of Health Psychology, University of Missouri School of Health Professions, Columbia, MO, United States; ^2^University of Missouri Thompson Center for Autism and Neurodevelopmental Disorders, Columbia, MO, United States; ^3^Department of Radiology, University of Missouri School of Medicine, Columbia, MO, United States; ^4^Department of Psychology, Marist College, Poughkeepsie, NY, United States; ^5^William and Nancy Thompson Endowed Chair in Radiology, Departments of Neurology and Psychological Sciences, University of Missouri, Columbia, MO, United States

**Keywords:** autism spectrum disorder, gastrointestinal disorders, problem behavior, internalizing symptoms, anxiety, depression

## Abstract

**Background:** Many individuals with autism spectrum disorder (ASD) have co-occurring gastrointestinal (GI) symptoms, but the etiology is poorly understood. These GI symptoms often coincide with problem behaviors and internalizing symptoms, which reduces the quality of life for these individuals.

**Methods:** This study examined the relationships among GI problems, problem behaviors, and internalizing symptoms in a sample of 340 children and adolescents with ASD who are patients at the University of Missouri Thompson Center for Autism & Neurodevelopmental Disorders.

**Results:** The majority of patients experienced constipation (65%), about half experienced stomachaches or stomach pain (47.9%), and others experienced nausea (23.2%) or diarrhea (29.7%). Young children with aggressive problem behaviors were 11.2% more likely to have co-occurring nausea; whereas, older children showed more complex relationships between internalizing symptoms and GI symptoms. Older children with greater anxiety symptoms were 11% more likely to experience constipation, but 9% less likely to experience stomachaches. Older children with greater withdrawn behavior were 10.9% more likely to experience stomachaches, but 8.7% less likely to experience constipation. Older children with greater somatic complaints were 11.4% more likely to experience nausea and 11.5% more likely to experience stomachaches.

**Conclusions:** Results suggest that the presentation of externalizing problem behavior and internalizing symptoms associated with GI problems differs between young children and older children with ASD. Therefore, behavior may have different relationships with GI symptoms at different ages, which may have implications for the treatment of and clinical approach to GI disturbances in ASD.

## Introduction

Autism spectrum disorder (ASD) is characterized by persistent deficits in social communication and social interaction across multiple contexts, as well as restricted and repetitive patterns of behavior, interests, and activities that occur early in life and cause clinically significant impairment (1). A variety of gastrointestinal (GI) issues commonly occur in ASD, including lower GI symptoms (i.e., constipation, diarrhea) and upper GI symptoms (i.e., nausea and vomiting, stomach aches and pains) (2–10), but the etiology is poorly understood.

Children with ASD have been shown to experience a range of GI symptoms, with the prevalence shown to be anywhere from 9 to 91% (2), which is likely due differences in assessment and context. However, it appears that many individuals with ASD suffer from constipation (2, 3, 11, 12). One association with GI issues in ASD may be the response to stress, since some individuals with ASD show an altered stress response (13) and recent research has shown connections between lower GI symptoms, sensory over-responsivity, and anxiety (14), as well as altered psychophysiological (11) and endocrine (12) responses to stress-inducing stimuli. These associations suggest that activation of the sympathetic nervous system and the hypothalamic-pituitary axis may be associated with GI disorders in ASD.

Consistent with the theory that stress is linked to GI symptoms in ASD, co-occurring internalizing symptoms, such as anxiety and depression, are common in ASD (15) and associated with GI symptoms as well. Heightened stress and anxiety, rigid-compulsive behavior, and sleep problems, have been shown to be associated with GI symptoms in ASD, especially constipation (6, 11, 12, 16–19). Children with ASD who experience GI symptoms also have co-occurring externalizing problems, as studies have shown that children with ASD with co-occurring GI symptoms had increased irritability when compared to those with ASD and no GI symptoms (5, 6).

The present study aimed to examine relationships among GI symptoms, externalizing problem behavior, and internalizing symptoms in a large sample of young children and older children and adolescents with ASD. We expected that the associations between GI symptoms and co-occurring conditions would be different across age groups. This study also aimed to determine which internalizing or externalizing problem behaviors would be associated with which GI symptoms. We hypothesized that regardless of age, anxiety would be associated with more constipation and diarrhea but less stomachaches and nausea due to the heightened stress responses association with lower GI symptoms.

## Methods

### Participants

This study included 340 children and adolescents with ASD ranging in age from 2 to 18 years old (*M* = 5.56, *SD* = 3.67) that are clinic patients at University of Missouri Thompson Center for Autism & Neurodevelopmental Disorders in Columbia, Missouri. All participants provided written informed consent in accordance with the Declaration of Helsinki, and the study was approved and carried out in accordance with the recommendations of the University of Missouri Health Sciences Institutional Review Board. Written informed consent was obtained from the parents/caregivers for all participants under the age of 18. Diagnosis of ASD was confirmed using the Autism Diagnostic Observation Schedule (20) or the Autism Diagnostic Interview—Revised (21). The sample included only participants from the database who had at least one GI symptom reported at the most recent clinic visit.

The sample was parsed into two age groups based on which version of the Child Behavior Checklist (CBCL) (22) the caregivers had completed for their child: younger (completed the CBCL for ages 2–5) or older (completed the CBCL for ages 6–18). The younger group consisted of 200 children (80% male), ranging in age from 2 to 5 (*M* = 3.03; *SD* = 1.07). The older group consisted of 140 children (77.1% male), ranging in age from 6 to 18 (*M* = 9.19; *SD* = 2.94).

### Measures

At each clinic visit, caregivers completed questionnaires about their child's developmental history and milestones. The responses to the questionnaires from the most recent clinic visit were obtained from the Thompson Center database, and the following variables were extracted and analyzed: dietary problems, nutrition problems, GI symptoms, and internalizing and externalizing symptoms.

#### Dietary Problems

Dietary problems were examined with the sum of 12 dichotomous caregiver-endorsed symptoms, with a total range from 0 to 12. The individual items included whether or not the child experienced the following: feeding issues in infancy, current feeding issues, picky eating, milk aversion, nonfood item cravings, food group aversion, food reactions, special diet, difficulty with solids, difficulty with liquids, lethargy, or dehydration.

#### Nutrition Problems

Nutrition problems were determined by the caregiver's response to the question, “Is the child's nutrition adequate?.” This variable was dummy coded, such that 0 = adequate nutrition and 1 = the child's nutrition was not adequate.

#### GI Symptoms

Caregivers completed a questionnaire about their concerns now or in the past about the following GI symptoms in their child: constipation, diarrhea, nausea or vomiting, and stomachaches or stomach pain. These were dichotomous variables, dummy coded such that 0 = no concerns, and 1 = concern. A total GI symptoms score was created by summing the four types of concerns, for a range of 1–4. In addition to the score for total GI problems, each individual GI symptom was considered separately in a subsequent analysis.

#### Internalizing and Externalizing Symptoms

Measures of internalizing and externalizing symptoms were derived from caregiver responses on the CBCL (22). The CBCL is a parent-report measure assessing behavioral and emotional symptoms in children. Items are rated on a 3-point Likert scale (0 = not true, 1 = somewhat or sometimes true, and 2 = very true or often true). Two versions of the CBCL are available based on the child's age. The CBCL has strong psychometric properties, including test-retest reliability, inter-rater agreement, and internal consistency (23–25).

### Covariates

Demographic covariates included age, gender, and household income, which were provided by caregivers at the time of assessment. Intelligence was assessed using a range of different assessments, each normalized to a mean of 100 and standard deviation of 15 (See Table 1).

**Table 1:** Statistical comparison of age groups on descriptive statistics.

	**Younger group (*****N*** **= 200)**			**Older group (*****N*** **= 140)**			**Statistical comparison**
	**Range**	**M**	**SD**	**Range**	**M**	**SD**	
Age in years	2–5	3.03	1.07	6–18	9.19	2.94	
IQ^#^							
Verbal	57–128	90.02	15.25	50–136	92.71	17.78	*t*_(153)_ = −0.989
Nonverbal	43–127	94.20	15.11	56–137	92.83	16.90	*t*_(182)_ = 0.578
Full scale	71–121	92.34	12.96	49–127	90.30	16.40	*t*_(168)_ = 0.869
Dietary problems	0–12	3.72	2.30	0–10	2.75	2.12	*t*_(224)_ = 3.182^**^
	***n***	**%**		***n***		**%**	**Statistical comparison**
Male	160	80		108		77.1	χ^2^(1) = 0.403
Race							χ^2^(6) = 8.168
Caucasian	165	82.5		111		79.3	
African	15	7.5		9		6.4	
Native	5	2.5		5		3.6	
Asian	4	2.0		1		0.7	
Unknown	11	5.5		14		10.0	
Household Income							χ^2^(4) = 9.985^*^
0–24,999	99	49.5		51		36.4	
25,000–49,999	50	25.0		29		20.7	
50,000–74,999	30	15.0		31		22.1	
75,000–99,999	8	4.0		11		7.9	
100,000+	10	5.0		12		8.6	
Missing	3	1.5		6		4.3	
Nutrition							χ^2^ (1) = 0.007
Adequate	121	60.5		89		63.6	
Not adequate	39	19.5		28		20	
Missing	40	20		23		16.4	
Taking GI meds							χ^2^(1) = 0.830
Yes	12	6		12		8.6	
No	188	94		128		91.4	
GI symptoms							*t*_(338)_ = 0.551
Constipation	129	64.5		92		65.7	
Diarrhea	65	32.5		36		25.7	
Nausea/vomiting	43	21.5		36		25.7	
Stomachache/pain	90	45		73		52.1	

# *The following intellectual assessments were used in the study, followed by the percentage that each assessment was used: Differential Ability Scales–Second Edition (26) (20.2%, General Conceptual Ability score), Wechsler Abbreviated Scale of Intelligence–Second Edition (27) (18.0%, Full Scale IQ score), Wechsler Intelligence Scale for Children–Fourth Edition (28) (5.9%, Full Scale IQ score), Wechsler Intelligence Scale for Children–Fifth Edition (29) (2.4%, Full Scale IQ score), Wechsler Preschool and Primary Scale of Intelligence–Third Edition (30) (0.3%, Full Scale IQ score), Wechsler Preschool and Primary Scale of Intelligence–Fourth Edition (31) (1.2%, Full Scale IQ score). A small group (11.7%) was administered a measure of nonverbal intelligence, the Leiter International Performance Scale–Third Edition (32); therefore, a Full-Scale IQ score was not available for this subsample. Intellectual testing could not be completed for many participants (40.3%) due to difficulties participating or understanding task demands*.

* *p < 0.05*;

** *p < 0.01*.

### Statistical Analyses

First, a simple bivariate correlation matrix among our variables was produced to determine which demographic or descriptive child and family covariates to include in our main analyses. Then, to evaluate the relationship between internalizing symptoms and externalizing problem behaviors for children with certain GI symptoms, we performed separate logistic regressions with the four GI symptoms as outcome variables for each age group. The primary predictors of interest were the internalizing and externalizing symptom subscales on the CBCL.

## Results

The majority of the sample experienced constipation (65%). About half of the children experienced stomachaches or stomach pain (47.9%), and others experienced nausea (23.2%) or diarrhea (29.7%). The average number of total GI symptoms was 1.66 (*SD* = 0.880), with a range from 1 to 4. The vast majority of the sample was not taking medications for GI symptoms (92.9%). However, over half of the children (53.2%) were taking at least one medication for other reasons (e.g., ADHD, aggression, anxiety symptoms, seizures, or sleep problems).

The two age groups did not differ significantly in gender composition [χ^2^(1) = 0.403, *p* = 0.308], nonverbal IQ [*t*_(182)_ = 0.578, *p* = 0.564], verbal IQ [*t*_(153)_ = −0.989, *p* = 0.324], or full scale IQ [*t*_(168)_ = 0.869, *p* = 0.386]. Families with younger children did have less household income [χ^2^(4) = 9.985, *p* = 0.041]. Younger and older children took similar amounts of GI medications [χ^2^(1) = 0.830, *p* = 0.242] and had similar rates of the four types of GI symptoms (*p*'s > 0.05). Older children were taking more total medications than younger children [*t*_(178.05)_ = −4.401, *p* < 0.001]. Younger children had significant more dietary problems [*t*_(224)_ = 3.182, *p* = 0.002], but similarly adequate nutrition as compared to older children [χ^2^ (1) = 0.007, *p* = 0.524]. See Table 1 for descriptive statistics by age group.

Covariates were determined by identifying any significant bivariate correlations for each age group. Therefore, in the younger age group, we controlled for dietary problems, total number of medications, GI medications, and nutrition problems. In the older age group, we controlled for gender and dietary problems.

### Do Internalizing or Externalizing Symptoms Predict GI Problems?

In younger children, aggressive problem behavior was a significant predictor of nausea, (*B* = 0.106, *SE* = 0.052, *p* < 0.05). Children with more aggression were 11.2% more likely to experience nausea problems.

In older children, several internalizing and externalizing symptoms were predictive of different GI problems. Older children with greater anxiety were 11% more likely to experience constipation problems (*B* = 0.094, *SE* = 0.048, *p* < 0.05), but 9% less likely to experience stomachaches (*B* = −0.099, *SE* = 0.050*, p* < 0.01.) Older children with greater withdrawn behavior were 10.9% more likely to experience stomachaches (*B* = 0.086, *SE* = 0.042, *p* < 0.05), but 8.7% less likely to experience constipation (*B* = −0.131, *SE* = 0.046, *p* < 0.01). Finally, older children with greater somatic complaints were 11.4% more likely to experience nausea (*B* = 0.130, *SE* = 0.050, *p* < 0.01) and 11.5% more likely to experience stomachaches (*B* = 0.138, *SE* = 0.049, *p* < 0.01). See Table 2 for logistic regression results.

**Table 2:** Logistic regression results for internalizing and externalizing symptoms predicting GI symptoms in younger (aged 2–5) and older (aged 6–18) children with ASD.

	**Younger group (*****N*** **= 200)**			**Older group (*****N*** **= 140)**		
**Predictor**	***B***	***SE B***	***e^***B***^***	***B***	***SE B***	***e^***B***^***
**CONSTIPATION**						
Withdrawn/depressed	–	–	–	−0.131^**^	0.046	0.877
Social problems	–	–	–	−0.070	0.050	0.932
Thought problems	–	–	–	0.007	0.016	1.007
Rule breaking	–	–	–	0.009	0.057	1.009
Emotionally reactive	0.042	0.065	1.043	–	–	–
Anxious/depressed	−0.030	0.083	0.971	0.094^*^	0.048	1.098
Somatic complaints	0.105	0.063	1.111	0.030	0.047	1.031
Withdrawn	−0.093	0.050	0.911	–	–	–
Sleep problems	−0.009	0.035	0.991	–	–	–
Attention	−0.003	0.056	0.997	0.076	0.041	1.079
Aggressive	−0.004	0.041	0.996	−0.068	0.048	0.935
**DIARRHEA**						
Withdrawn/depressed	–	–	–	0.033	0.036	1.034
Social problems	–	–	–	0.049	0.044	1.050
Thought problems	–	–	–	−0.003	0.007	0.997
Emotionally reactive	0.025	0.056	1.026	–	–	–
Anxious/depressed	−0.047	0.076	0.954	−0.074	0.042	0.928
Somatic complaints	−0.035	0.053	0.966	−0.001	0.043	0.999
Withdrawn	0.020	0.044	1.020	–	–	–
Sleep problems	0.012	0.032	1.012	–	–	–
Attention	−0.027	0.055	0.973	−0.019	0.033	0.982
Aggressive	0.039	0.036	1.040	0.059	0.041	1.061
**NAUSEA**						
Withdrawn/depressed	–	–	–	0.033	0.039	1.034
Social problems	–	–	–	−0.036	0.045	0.964
Thought problems	–	–	–	−0.005	0.012	0.995
Emotionally reactive	0.006	0.067	1.006	–	–	–
Anxious/depressed	−0.167	0.110	0.846	−0.005	0.044	0.995
Somatic complaints	0.084	0.062	1.087	0.130^**^	0.050	1.139
Withdrawn	−0.009	0.054	0.991	–	–	–
Sleep Problems	−0.065	0.048	0.937	–	–	–
Attention	−0.045	0.063	0.956	0.028	0.037	1.029
Aggressive	0.106^*^	0.052	1.112	−0.051	0.045	0.950
**STOMACHACHES**						
Withdrawn/depressed	–	–	–	0.086^*^	0.042	1.090
Social problems	–	–	–	−0.009	0.050	0.991
Thought problems	–	–	–	0.005	0.011	1.005
Rule breaking	–	–	–	0.094	0.058	1.098
Emotionally reactive	0.053	0.057	1.054	–	–	–
Anxious/depressed	0.011	0.072	1.011	−0.099^*^	0.050	0.906
Somatic Complaints	0.020	0.048	1.020	0.138^**^	0.049	1.148
Withdrawn	−0.018	0.040	0.982	–	–	–
Sleep problems	0.011	0.031	1.012	–	–	–
Attention	−0.033	0.047	0.967	−0.009	0.035	0.991
Aggressive	−0.007	0.033	0.993	−0.028	0.047	0.973

*Controls for the younger group are GI medications, total medications, dietary problems, and nutrition problems (omitted from the table). Controls for the older group are gender and dietary problems (omitted from the table)*.

*B = The unstandardized beta value represents the slope of the line between the predictor variable and the dependent variable. For example, for older children, for every one unit increase in the Withdrawn/Depressed subscale, Constipation problems decrease by 0.131 units*.

*SE B = The standard error for the unstandardized beta describes the dispersion of the distribution around the regression line. For example, a larger number indicates the scores are more spread out from the regression line and there is more error in prediction*.

*e^B^ = The exponential B is the odds ratio for the predictor. For example, for older children, for every one unit increase in the Withdrawn/Depressed subscale, an individual is 0.877 times more likely to have Constipation than not have Constipation*.

* *p < 0.05*;

** *p < 0.01*.

## Discussion

In the current study, constipation accounted for 65% of the GI symptoms in a sample of 340 children and adolescents with ASD. This finding corroborates previous reports in the literature showing that constipation accounts for a significant amount of GI complaints in ASD (2, 11, 12). Regarding externalizing problem behavior, presence of aggressive behavior was associated with nausea in children aged 2–5, suggesting that aggression may be an indicator of nausea in young children in this this population. However, no other GI problems were significantly associated with problem behavior and internalizing symptoms in young children with ASD. One explanation for this relationship may be that young children with ASD who are non-verbal use aggression as a means of communicating somatic complaints, such as internal abdominal pain and GI discomfort (2).

Children and adolescents aged 6–18 revealed associations between internalizing symptoms but not externalizing problem behaviors, and GI symptoms. In the older group, presence of anxiety conferred an 11% increase in the presence of constipation symptoms, which corroborates recent reports on an association between anxiety and lower GI tract symptoms (11, 12). Given these findings, future research may wish to examine the effects of behavioral and/or pharmacological stress and anxiety reductions on GI symptoms in ASD. Interestingly, withdrawn and depressed behavior was associated with an 11% increase in stomachaches, but a 9% decrease in constipation. Previous research has also shown a relationship between upper GI tract symptoms, including stomachaches, and depression in ASD, suggesting that depression and anxiety may place an individual with ASD at heightened risk for GI disturbance, but in different manners. Taken together, it is possible that GI disorders and behavioral problems are related in ASD as a means of communicating their discomfort given the core language deficits in those with ASD. Further research is needed to disentangle the relationship between GI symptoms, anxiety, and depression to determine how these disorders cluster with upper or lower GI tract disorders in ASD.

This study has a number of limitations that should be considered alongside of the results. First, the sample is largely male and Caucasian in both age groups, making it difficult to generalize the findings to females and non-Caucasian individuals with ASD. Second, the GI symptoms were from caregiver reports and were not further assessed with a gastroenterological evaluation. Future studies of GI disorders in ASD should aim to utilize standardized measurements to assess GI symptoms, especially those which are ASD-specific (33, 34). Last, participants included in this study were selected based on having at least one GI symptom, so the results may not be representative of the general population of those with ASD. Furthermore, the sample of participants was from one clinic in the Midwestern United States, and so data from other clinics in other regions are needed in order to generalize these findings to the larger ASD population. In sum, this study provides further evidence of the relationship between co-occurring conditions and GI symptoms in ASD, and highlights the need to evaluate age-related developmental differences in the associations among these symptoms, which may prove to be important when developing GI treatments for those with ASD across the lifespan.

## Ethics Statement

This study was carried out in accordance with the recommendations of the Health Sciences Institutional Review Board at the University of Missouri with written informed consent from all subjects. All subjects gave written informed consent in accordance with the Declaration of Helsinki. The protocol was approved by the Health Sciences Institutional Review Board at the University of Missouri.

## Author Contributions

BF and DB conceived the aforementioned studies conducted at the University of Missouri Thompson Center for Autism & Neurodevelopmental Disorders. DB provided expertise on ASD and GI disorders in ASD. KD provided the statistical analysis for the study. NT maintained the database from which the data for this study were obtained. All authors contributed to the development of the manuscript and approved the final version that was submitted for review.

### Conflict of Interest Statement

The authors declare that the research was conducted in the absence of any commercial or financial relationships that could be construed as a potential conflict of interest.
